# A Prolyl-Hydroxylase Inhibitor, Ethyl-3,4-Dihydroxybenzoate, Induces Cell Autophagy and Apoptosis in Esophageal Squamous Cell Carcinoma Cells via Up-Regulation of BNIP3 and N-myc Downstream-Regulated Gene-1

**DOI:** 10.1371/journal.pone.0107204

**Published:** 2014-09-18

**Authors:** Bo Han, Wei Li, Yulin Sun, Lanping Zhou, Yang Xu, Xiaohang Zhao

**Affiliations:** State Key Laboratory of Molecular Oncology, Cancer Institute & Hospital, Chinese Academy of Medical Sciences & Peking Union Medical College, Beijing, China; Virginia Commonwealth University, United States of America

## Abstract

The protocatechuic acid ethyl ester ethyl-3,4-dihydroxybenzoate is an antioxidant found in the testa of peanut seeds. Previous studies have shown that ethyl-3,4-dihydroxybenzoate can effectively reduce breast cancer cell metastasis by inhibiting prolyl-hydroxylase. In this study, we investigated the cytotoxic effect of ethyl-3,4-dihydroxybenzoate on esophageal squamous cell carcinoma cells in vitro and identified key regulators of ethyl-3,4-dihydroxybenzoate-induced esophageal cancer cell death through transcription expression profiling. Using flow cytometry analysis, we found that ethyl-3,4-dihydroxybenzoate induced S phase accumulation, a loss in mitochondrial membrane permeabilization, and caspase-dependent apoptosis. Moreover, an expression profile analysis identified 46 up- and 9 down-regulated genes in esophageal cancer KYSE 170 cells treated with ethyl-3,4-dihydroxybenzoate. These differentially expressed genes are involved in several signaling pathways associated with cell cycle regulation and cellular metabolism. Consistent with the expression profile results, the transcriptional and protein expression levels of candidate genes *NDRG1*, *BNIP3, AKR1C1, CCNG2* and *VEGFA* were found to be significantly increased in treated KYSE 170 cells by reverse-transcription PCR and western blot analysis. We also found that protein levels of hypoxia-inducible factor-1α, BNIP3, Beclin and NDRG1 were increased and that enriched expression of BNIP3 and Beclin caused autophagy mediated by microtubule-associated protein 1 light chain 3 in the treated cells. Autophagy and apoptosis were activated together in esophageal cancer cells after exposed to ethyl-3,4-dihydroxybenzoate. Furthermore, knock-down of NDRG1 expression by siRNA significantly attenuated apoptosis in the cancer cells, implying that NDRG1 may be required for ethyl-3,4-dihydroxybenzoate-induced apoptosis. Together, these results suggest that the cytotoxic effects of ethyl-3,4-dihydroxybenzoate were mediated by the up-regulation of NDRG1, BNIP3, Beclin and hypoxia-inducible factor-1α, initiating BNIP3 and Beclin mediated autophagy at an early stage and ultimately resulting in esophageal cancer cell apoptosis.

## Introduction

Esophageal cancer is the sixth leading cause of cancer-related death worldwide and ranks as the fourth most common cause of cancer-related death in China based on the GLOBOCAN 2008 estimates (http://globocan.iarc.fr/) [1], [2]. There are two main subtypes of esophageal cancer, esophageal squamous cell carcinoma (ESCC) and esophageal adenocarcinoma, with ESCC being the most frequent type of esophageal malignancy. Patients are typically already in the advanced stages of the disease when first diagnosed; as a result, the effects of treatment are poor [3], [4], and the 5-year survival rate is less than 19% [5], [6].

**Figure 1:**
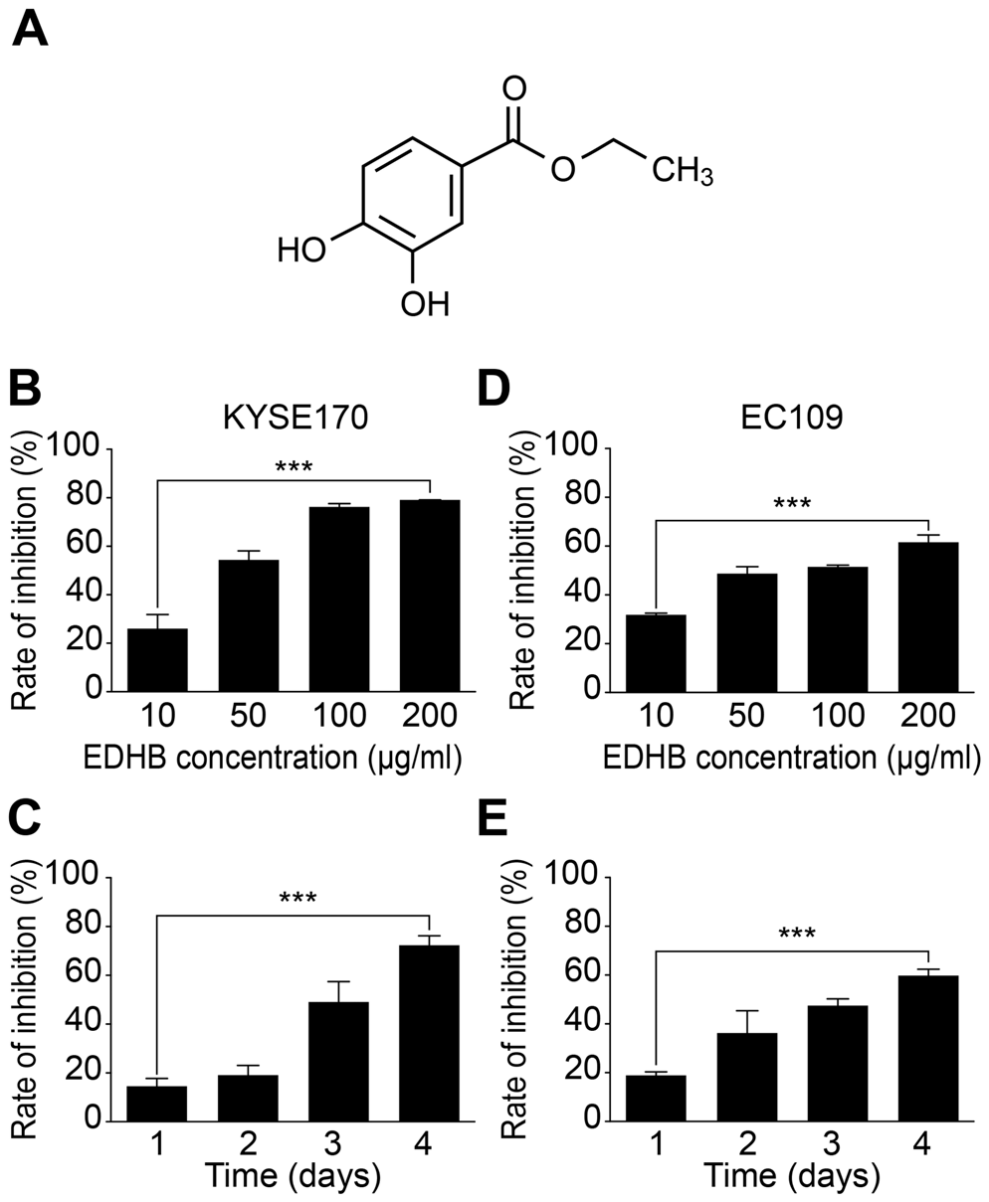
EDHB inhibited the proliferation of ESCC cells. (A) The chemical structure of EDHB. (B) KYSE 170 cells were treated with various concentrations of EDHB for 3 days, and the inhibition of cell proliferation was determined with the WST-8 assay. (C) KYSE 170 cells were treated with 50 µg/ml EDHB for 4 days, and the inhibition of cell proliferation was determined with the WST-8 assay. (D) EC109 cells were treated with various concentrations of EDHB for 3 days, and the inhibition of cell proliferation was determined with the WST-8 assay. (E) EC109 cells were treated with 50 µg/ml EDHB for 4 days, and the inhibition of cell proliferation was determined with the WST-8 assay. ***, *P*<0.001.

Esophageal cancer chemoprevention studies have suggested that some natural foods, such as strawberries, blueberries, and black raspberries, and chemical monomers are associated with a reversal of esophageal dysplasia [7]. Recently, chemoprevention studies based on a phase II clinical trial in esophageal cancer showed that strawberries could significantly reduce the histological grade of precancerous lesions of the esophagus [8]. The mechanism underlying these effects may be associated with the inhibition of cell proliferation, inflammation, and tumor angiogenesis [9], [10]. Another trial showed that nutritional intervention significantly prevented ESCC development after dietary supplementation with selenium, vitamin E, and beta-carotene [11]. In addition, combined treatment with the COX-2 inhibitor L-748706 and an anti-inflammatory drug, such as piroxicam, inhibited the occurrence and development of esophageal cancer [12]. Phenolic compounds, which act as chemopreventive agents are widely found in fruits and vegetables and have strong antioxidant effects, inducing apoptotic cell death as well as inhibiting tumor growth [13], [14], [15]. Polyphenols in tea leaves were also shown to significantly inhibit mouse skin tumor growth induced by treatment with 7,12-dimethylbenz[α]anthracene [16]. Ethyl-3,4-dihydroxybenzoate (EDHB) is a polyphenolic compound (Figure 1A) present in many plants, such as peanut seed testa, and is commonly used as a food additive. EDHB contains reducible polyphenol hydroxyl groups and exhibits antioxidant activity [17]. Recent studies have shown that EDHB acts as an analog of the substrate α-ketoglutarate and competes for prolyl-hydroxylase activity, thus acting as an inhibitor and effectively inhibiting collagen synthesis and breast cancer metastasis [18]. In addition, *in vitro* and animal studies in a cerebral ischemic rat model have revealed that EDHB shows increased protective effects and improves rat behavior by inhibiting free radical damage [19]. However, whether EDHB can inhibit esophageal cancer cell growth and the possible underlying molecular mechanisms remain unknown.

In this study, we investigated the effects of EDHB on cell proliferation and cell death in esophageal cancer cells. Moreover, we also investigated the mechanisms of EDHB-induced cell death. Interestingly, we found that EDHB treatment not only led to apoptosis but also autophagic cell death via the up-regulation of BCL-2/EIB-19K-interacting protein 3 (BNIP3) and Beclin and the down-regulation of BCL-2 in the esophageal cancer KYSE 170 cells. The microarray results identified a panel of differentially expressed genes, including BNIP3, Beclin and N-myc downstream-regulated gene-1 (NDRG1). The increased expression of BNIP3 and Beclin initiated autophagy at an early stage, which was mediated by microtubule-associated protein 1 light chain 3 (LC3) in KYSE 170 cells after treatment with EDHB for 12 h, ultimately result in the apoptosis of esophageal cancer cells. NDRG1 knock-down with a siRNA significantly attenuated apoptosis, implying that NDRG1 may be required for EDHB-induced apoptosis. Overall, our results lead to better understand the mechanism of action of EDHB, and suggest that EDHB may be of potential therapeutic value for the treatment of esophageal cancer.

## Materials and Methods

### Reagents

The EDHB chemical compound was purchased from Sigma-Aldrich (St. Louis, MO) and dissolved in ethanol for biochemical assays. Anti-NDRG1 and anti-BINP3 antibodies were purchased from Abcam (Abcam, MA). Anti-caspase-3 and anti-caspase-9 antibodies were purchased from Enzo Life Sciences (Life Sciences, NY). Anti-HIFα, anti-Beclin, anti-ATG12 and anti-LC3 antibodies were purchased from Cell Signaling Technologies (Danver, MA). Anti-AKR1C1, anti-Bcl-2, anti-p53, anti-TOM20 and anti-p21 antibodies were purchased from Santa Cruz Biotechnology (Santa Cruz, CA).

### Cell lines and cell cultures

The human ESCC cell line KYSE 170 was a generous gift from Dr. Shimada Y, (Kyoto University, Japan) [20]. The EC109 cell line was purchased from the National Platform of Experimental Cell Resource for Sci-Tech (http://cellresource.cn/) (Beijing, China). These cells were cultured in complete RPMI 1640 medium, supplemented with 10% fetal bovine serum (FBS), 100 U/ml penicillin and 100 µg/ml streptomycin, and maintained at 37°C and 5% CO_2_.

### Cell proliferation assay

WST-8 assays for cell proliferation were performed using the Cell Counting Kit-8 (CCK-8) (Dojindo, Japan). The cells were plated into 96-well plates and treated with varying concentrations (0, 10, 50, 100, and 200 µg/ml) of EDHB for 24, 48, 72, and 96 h. Untreated cells were used as a negative control. Culture medium (100 µl) was added to each well, and the cells were then incubated with 10 µl of WST-8 in the culture medium for 3 h. The absorbance values were detected at 450 nm using an enzyme-linked immunosorbent assay (ELISA) reader.

### Detection of mitochondrial membrane permeability

Cells (4×10^5^) were plated in 6-well plates and incubated overnight. The cells were treated with 50 µg/ml EDHB for 24, 48, and 72 h and then stained with the mitochondria-specific fluorescent probe MitoTracker Red (10 µM). The intensity of red fluorescence in the cells was detected using flow cytometry (BD Biosciences, San Jose, CA).

### Analysis of cell cycle and apoptosis

Cells (4×10^5^) were plated in 6-well plates and cultured overnight. The cells were treated with varying concentrations of EDHB for 24, 48, and 72 h and harvested. A cell apoptosis detection reagent kit (Biosea Biotechnology, Beijing, China) was used according to the detailed procedure provided in the instruction manual. The cells were stained with Annexin-V and propidium iodide (PI), and the fluorescence intensity was detected using flow cytometry. Cell cycle detection reagent kit (4A Biotech, Beijing, China) was used according to the manufacturer's instructions. The cells were stained with PI (4A Biotech, Beijing, China) and the DNA content during different phases of the cell cycle was detected using flow cytometry (BD Biosciences, San Jose, CA).

### RNA extraction

Cells were treated with 50 µg/ml EDHB for 12 h, and aliquots of 1×10^6^ to 1×10^7^ cells from each sample were collected. Total RNA was extracted using TRIzol reagent and further purified using Qiagen RNeasy Mini Kit (Qiagen, Hilden, Germany) according to the manufacturers' instructions. The RNA concentrations were quantified using an ultraviolet Spectrophotometer (NanoDrop Technologies, ND-1000), and the quality of RNA was assessed by formaldehyde agarose gel electrophoresis.

### Microarray experiments

A 200 ng sample of total RNA was used to synthesize double-stranded cDNA, and the modified nucleotide, biotin-UTP, was incorporated into the aRNA synthesized by the MessageAm Premier RNA Amplification Kit (Life Technologies, Grand Island, NY). The biotinylated aRNA was fragmented into strands of 35–200 bases according to the Affymetrix' protocol prior to hybridization. After measuring the concentration and verifying the fragment lengths, the biotinylated aRNAs were hybridized to the Human Genome U133 Plus 2.0 Array (Affymetrix, Santa Clara, CA). Hybridization was performed at 45°C for 16 h with constant rotation at 60 rpm in a GeneChip Hybridization Oven. After washing and staining using an automatic Affymetrix Fluidics Station 450, the GeneChip arrays were scanned using an Affymetrix GeneChip Scanner 3000 7G scanner.

The GeneChip Human Genome U133 Plus 2.0 Array contains 47,000 transcripts, which represented 38,500 human genes. The database sources include GenBank, dbEST, and RefSeq. GEO series number is GSE58024.

### Analysis of microarray data

The scanned images were first assessed by visual inspection and were then analyzed to generate CEL files using the default setting of Affymetrix GeneChip Command Console 3.2 (AGCC) software. The raw data were normalized and summarized using the Affymetrix Microarray Suite 5.0 (MAS5) and the Robust Multi-array Average (RMA) algorithm. Quality controls from Affymetrix to ensure the reliability of the results were included according to the manufacturer's instructions.

For comparison analysis, we applied a two class unpaired method using the Significant Analysis of Microarray software (SAM, version 3.02) to identify significantly differentially expressed genes between the sample and control groups. Genes were significantly differentially expressed at a fold change value of 2.0 and q value <5% as cutoffs, according to the SAM output results. Hierarchical clustering using the average linkage method was performed with Cluster 3.0 software, and the cluster result was visualized with the Treeview program.

### Real-time PCR

Total RNA was extracted from KYSE 170 cells. After the reverse transcription of total RNA, PCR was performed as follows: initial denaturation at 94°C for 2 min, and 30 cycles of 95°C for 10 s and 58°C for 30 s. Each experiment was performed in triplicate using the TAqMan 7900 (ABI) real-time PCR machine and the QuantiFast SYBR Green PCR Kit (Qiagen, Valencia, CA) according to the manufacturer's instructions. Gene-specific primers for the selected genes were as follows: *NDRG1*: forward 5′GTAAGTCAGCCACTGGGACC, reverse 5′ACGAGTCATTGCCTCTCACG; *CCNG2*: forward 5′ AGGTGGCTTTCCCATGACTG, reverse: AACGGGGGTAAGGATGAGGA; *VEGFA*: forward 5′ TCTCCCTGATCGGTGACAGT, reverse 5′ AAGGAATGTGTGCTGGGGAG; *BNIP3*: forward 5′ CCTCCACCAGCACCTTTTGA, reverse 5′ CCACCCCAGGATCTAACAGC; and *AKR1C1*: forward 5′ ATGGTGACACAGAGGATGGCT, reverse 5′ TTTGCTGTAGCTTGCTGAAATCAC.

### Immunoprecipitation and western blot analysis

Immunoprecipitation of BNIP3 and LC3 was performed according to a previously described protocol [21]. Briefly, cells cultured in 10-cm dishes were lysed for 30 min on ice in lysis buffer containing 1% (w/v) Triton X-100, 150 mM NaCl, 30 mM Tris-HCl (pH 7.5) and protease inhibitors (Roche, Germany). The lysates were sonicated and centrifuged at 10,000 *g* for 15 min at 4°C. One milligram of extracted protein in lysis buffer was incubated overnight with 10 µg rabbit anti-BNIP3, anti-LC3 antibodies or relevant IgG as control followed by incubation with 20 µl Dynabeads Protein G (Invitrogen, USA) for 2 h at 4°C and three washes with lysis buffer. The beads were directly boiled in 1% SDS loading buffer for 5 min.

Proteins were extracted from KYSE 170 cells. After treatment with 50 µg/ml EDHB, the cells were lysed with RIPA buffer (50 mM Tris-HCl, pH 8.0, with 150 mM NaCl, 1.0% Igepal CA-630 (NP-40), 0.5% sodium deoxychlorate, and 0.1% SDS) containing a protease inhibitor (500 mM phenylmethylsulfonyl fluoride) for 30 min. A total of 30 µg of the protein samples was electrophoresed on a 10% SDS-PAGE gel and transferred to a polyvinylidene difluoride membrane. The membranes were blocked for 1 h with 5% (w/v) nonfat dry milk in PBS and incubated with primary antibodies at 4°C overnight. Western blot analysis was performed using the following antibodies: anti-caspase-9, anti-caspase-3, anti-Fos, anti-BNIP3, anti-Beclin, anti-Bcl2, anti-LC3, anti-p53, etc. Proteins were detected with secondary antibodies conjugated to horseradish peroxidase and visualized with the Renaissance Plus reagent (Life Technologies, Grand Island, NY).

### Subcellular extraction of adherent tissue culture cells

Prior to extraction, the buffers were mixed well by vortexing. During the extraction procedure, the I–III and Benzonase buffers were kept on ice, and Buffer IV and Protease Inhibitor Cocktail were kept at room temperature. The different cellular fractions were extracted using ProteoExtract Subcellular Proteome Extraction Kit (Calbiochem, Darmstadt, Germany) according to the manufacturer's instructions.

### siRNA transfection

Prior to transfection, cells were plated in 6-well plates and allowed to reach 80% confluence for transfection. After culture for 12–16 h, 5 µl of Lipofectamine liposomes and 5 µl of siRNA (sequences: 5′-CCUCCUGCAAGAGUUUGAUTT -3′ and 5′-AUCAAACUCUUGCAGGAGGTT-3′) (Life Technologies, Carlsbad, California, USA) were separately pre-incubated in 250 µl of Opti-MEM culture medium for 5 min. These two solutions were then mixed and incubated at room temperature for 10 min. The cells were washed twice with PBS and pre-incubated in 2 ml of serum free culture medium. The mixture containing the siRNA and liposomes was then added to the culture medium, and the medium was replaced with culture medium containing 10% FBS after 6 h. After 48 h, the cells were harvested, and the transfection efficiency was evaluated using western blot analysis.

### Immunofluorescence staining

Cells grown on glass coverslips were fixed in 4% paraformaldehyde for 30 min, and rinsed with PBS. The cells were then permeabilized with 0.1% Triton X-100, rinsed and incubated with PBS containing 2% BSA for 30 min. The cells were incubated for 1 h at room temperature with the appropriate primary antibodies, rinsed several times and incubated at room temperature for 30 min with the relevant fluorescent secondary antibodies. Fluorescence images were captured with a Nikon ECLIPSE 80i microscope.

### Statistical methods

The data were analyzed using SPSS16 software. Analysis of variance (ANOVA) and *t*-tests were performed. *P*<0.05 indicated statistically significant differences.

## Results

### Inhibitory effects of EDHB on esophageal cancer cell proliferation

To determine whether EDHB could reduce the growth of esophageal cancer cells *in vitro*, we treated the KYSE 170 and EC109 cells with various concentrations of EDHB. After 72 h of treatment, EDHB reduced cell viability by more than 50% in the treated compared to the untreated cells. The IC50 values were 48.3 µg/ml for KYSE 170 cells and 72.1 µg/ml for EC109 cells. The effect of EDHB was dose-dependent, as increasing concentrations of EDHB further decreased the number of viable cells (Figure 1B and D). In addition, the effect of EDHB was time-dependent (Figure 1C and E). We also observed dose- and time-dependent effects of EDHB in EC109 cells. Therefore, we concluded that EDHB was capable of inhibiting the proliferation of ESCC cells.

### EDHB induced cell cycle arrest in KYSE 170 cells

**Figure 2:**
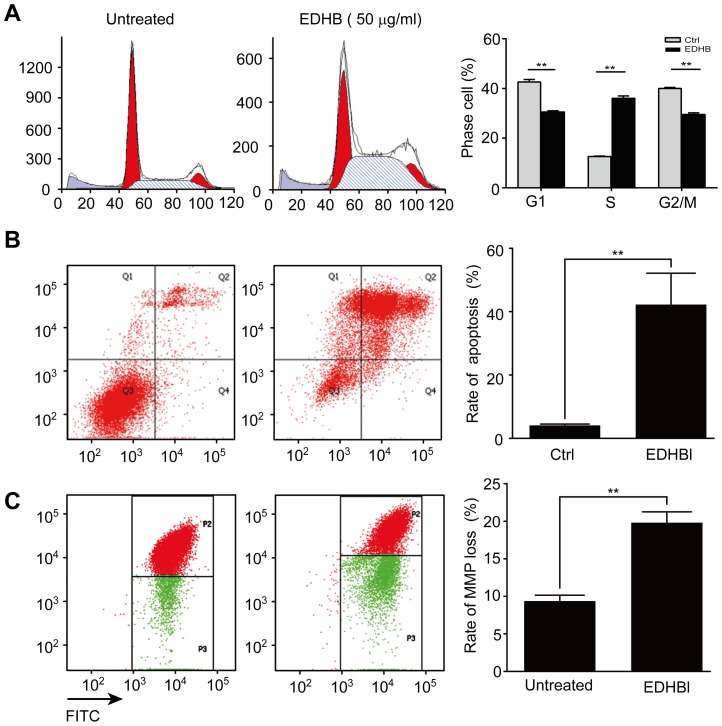
EDHB induced changes in cellular morphology. KYSE170 cells were treated with EDHB for 48 h and 72 h. (A) The cell cycle analysis was determined at the 48 h time point; the cell cycle profiles are shown. The histogram of statistical analysis on the right is the percentage of S phase cells. (B) Apoptosis was analyzed at the 72 h time point by Annexin-V-PI staining using flow cytometry. The histogram of statistical analysis on the right is the percentage of cell death. (C) The mitochondrial membrane permeabilization was measured at the 48 h time point using flow cytometry. The histogram of statistical analysis on the right is the rate of mitochondrial membrane permeabilization loss. Ctrl, EDHB untreated control; **, *P*<0.05.

As EDHB reduced esophageal cancer cell growth, we next determined whether this decrease was due to alterations in the cell cycle. The results showed that EDHB induced cell cycle arrest in KYSE 170 cells after 2 days of treatment, as detected by PI staining using flow cytometry. As shown in Figure 2A, KYSE 170 cells were treated with 50 µg/ml EDHB for 2 days, and the ratios of cells in different phases of the cell cycle were detected using flow cytometry. Compared to the control group, EDHB treatment resulted in an accumulation of KYSE 170 in S phase. A statistical analysis of the cell ratio in S phase revealed that EDHB treatment significantly increased the percentage of cells in S phase and led to cell cycle arrest.

### EDHB induced apoptosis and loss of mitochondrial membrane permeabilization in KYSE 170 cells

To determine whether ESCC cells undergo apoptosis upon treatment with EDHB, KYSE 170 cells were treated with EDHB for 72 h and measured the apoptotic cells by Annexin V-IP staining. The percentage of apoptotic KYSE 170 cells in the 50 µg/ml EDHB-treated group was 40% (Figure 2B), and a statistical analysis confirmed that the difference between the treated and control group was significant (*P*<0.05). Taken together, these results suggest that EDHB reduced cell growth by inducing cell cycle arrest and apoptosis.

To investigate whether the observed EDHB-induced cell apoptosis was associated with mitochondrial dysfunction, we analyzed mitochondrial membrane permeabilization in KYSE 170 cells by staining the cells with MitoTracker, a mitochondria-sensitive dye, for flow cytometry analysis. As shown in Figure 2C, the mitochondrial membrane permeabilization of KYSE 170 cells was significantly decreased after treatment with 50 µg/ml EDHB for 2 days. The statistical analysis revealed that the difference between the treated and untreated groups was significant (*P*<0.05), indicating that EDHB induced cell apoptosis through mitochondrial dysfunction in KYSE 170 cells.

### Transcriptional expression profile of KYSE 170 cells after treatment with EDHB

We next evaluated changes in the gene expression profile of KYSE 170 cells after 12 h treatment with EDHB. Each experiment included two biological replicates. As shown in Figure S1 A, the following criteria were used to determine differential gene expression: genes with fold changes>2 were determined to be significantly up-regulated, and genes with fold changes <0.5 were determined to be significantly down-regulated. Group 1 contained 67 genes with increased expression and 24 genes with decreased expression. Group 2 contained 107 genes with increased expression and 33 genes with decreased expression. There were 43 genes with increased expression in both groups and 9 genes with decreased expression in both groups.

Based on the differentially expressed genes, as analyzed using the Kyoto Encyclopedia of Genes and Genomes (KEGG) and BIOCARD pathways, the 43 differentially up-regulated genes in both groups were subjected to a Gene Ontology analysis. As shown in Figure S1 B, 13 cytoplasmic proteins, 10 nuclear proteins, and 7 membrane proteins were found to be involved in various biological processes, such as mitosis, amino acid biosynthesis, hypoxia responses, cell cycle regulation, and prostaglandin metabolism. A gene correlation analysis was performed with the 43 differentially up-regulated genes, and *NDRG1*, *CCNG2*, *VEGF*, *BNIP3*, and *AKR1C1* were subjected to further validation (Figure S1 C).

### Validation of candidate up-regulated genes by real-time PCR and western blot analysis

**Figure 3:**
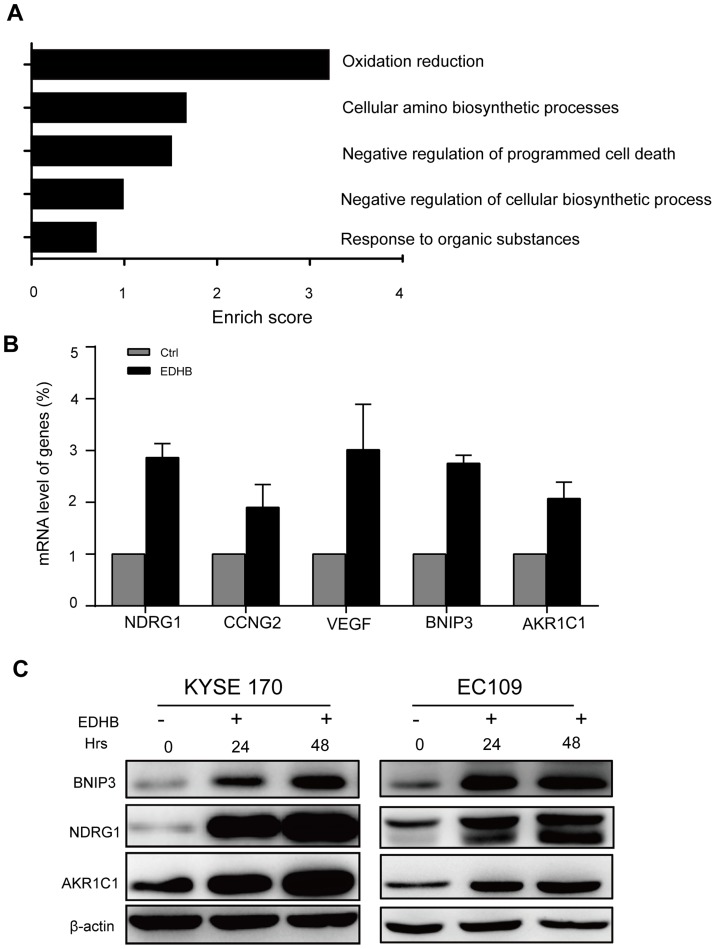
Candidate gene validation using real-time PCR and western blot analysis. (A) Enriched score analysis of differentially expressed genes. (B) Candidate genes were validated using real-time PCR. (C) Candidate proteins including NDRG1, BNIP3 and AKR1C1 were validated by western blot analysis in KYSE170 and EC109 cells. Ctrl, control; Hrs, hours.

Based on the analysis by the DAVID database, the enrichment analysis of cellular processes suggested that the identified genes are primarily involved in biological processes, including response to organic substances, negative regulation of cellular biosynthetic processes, regulation of programmed cell death, cellular amino acid biosynthetic processes, and oxidative stress responses (Figure 3A). Changes in *NDRG1*, *CCNG2*, *VEGF*, *BNIP3*, and *AKR1C1* transcriptional and protein expression were next validated by real-time PCR and western blot analysis, respectively. KYSE 170 cells were treated with EDHB for 12 h, and the gene expression results were validated using real-time PCR. As shown in Figure 3B, the transcript levels of the *NDRG1*, *CCNG2*, *VEGF*, *BNIP3*, and *AKR1C1* genes demonstrated fold increases of 2.9, 2.1, 2.6, 2.7, and 2.1, respectively, upon EDHB treatment.

To confirm that the observed changes in mRNA expression reflected changes in protein expression, the protein expression levels of three candidate genes, *NDRG1*, *BNIP3*, and *AKR1C1*, were next examined by a western blot analysis. Consistent with the real-time PCR results, the protein expression levels of NDRG1, BNIP3, and AKR1C1 were increased in both KYSE 170 and EC109 cells after treatment with EDHB for 24 and 48 h (Figures 3C). These results indicated that EDHB induced alterations in *NDRG1*, *BNIP3*, and *AKR1C1* gene and protein expression.

### EDHB promoted apoptosis-related protein alterations

**Figure 4:**
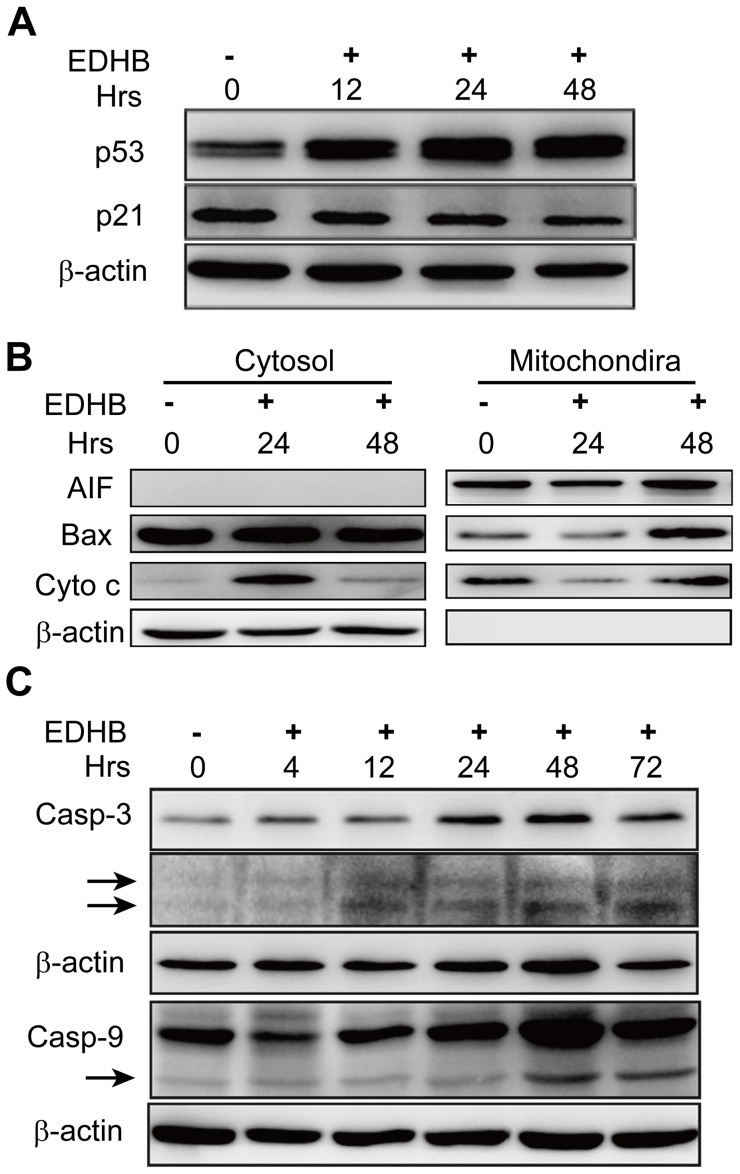
Verification of apoptotic protein expression by western blot analysis. (A) KYSE 170 cells were treated with 50 µg/ml EDHB for 0 h, 12 h 24 h and 48 h, and p53 and p21 protein expression was analyzed by western blot analysis. (B) KYSE170 cells were treated with 50 µg/ml EDHB for 24 or 48 h. Mitochondrial and cytosolic fractions were isolated from the treated cells and analyzed for the indicated proteins by western blotting. AIF was used as a control for the mitochondrial fraction and loading. (C) KYSE 170 cells were treated with 50 µg/ml EDHB for 0 h, 4 h, 12 h, 24 h, 48 h and 72 h, caspase-3 and caspase-9 protein expression was analyzed by western blot analysis. Hrs, hours.

Our results indicated that EDHB induced KYSE 170 cell apoptosis. To examine the molecular mechanism underlying this EDHB-mediated apoptosis, we analyzed p53, p21, caspase-9 and caspase-3 expression by western blotting. In the EDHB-treated KYSE 170 cells, the expression level of p53 was markedly increased, whereas p21 expression gradually decreased (Figure 4A).

These results showed that EDHB induced KYSE 170 cell death through p53, caspase-9, and caspase-3 activation, ultimately leading to apoptosis. Because both Bax translocation to and cytochrome c release from the mitochondria are thought to be the major mechanism of caspase-9 activation, we further analyzed these factors after treatment with EDHB for 24 h and 48 h. Cytosolic and mitochondrial fractions were isolated and analyzed for the expression of Bax and cytochrome c by western blotting. Bax was greatly enriched in the mitochondrial fraction and cytochrome c was significantly accumulated in the cytosolic fractions of KYSE170 cells following EDHB treatment for 24 h and 48 h (Figure. 4B). In addition, the activated cleaved fragments of caspase-9 and caspase-3 were significantly increased in a time-dependent manner (Figure 4C). Taken together, the results indicated that EDHB induced apoptosis in KYSE 170 cells via the intrinsic mitochondrial pathway.

### Up-regulation of HIF, BNIP3 and Beclin proteins contribute autophagy induced by EDHB

**Figure 5:**
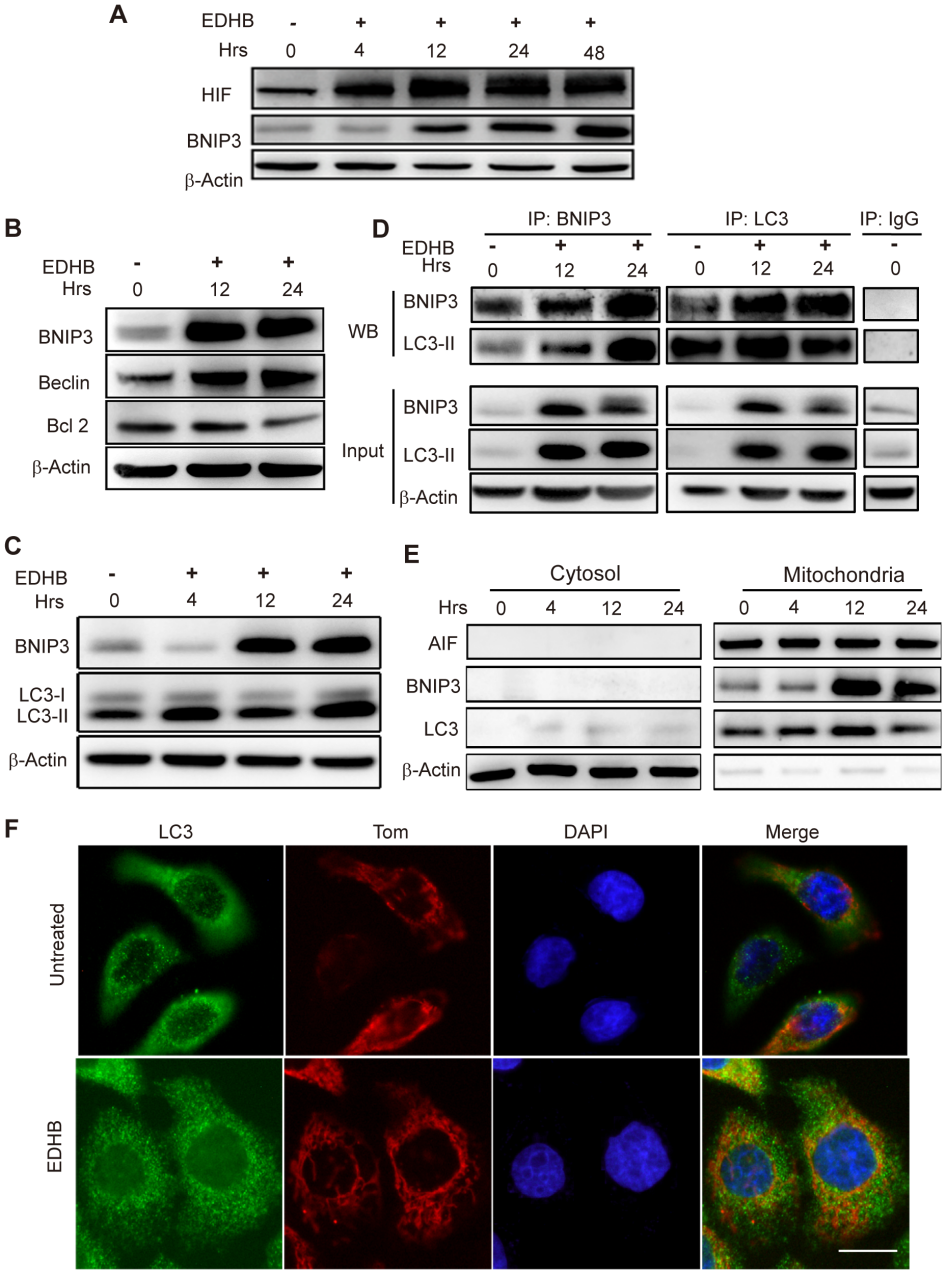
Up-regulation of HIF, BNIP3 and Beclin proteins contribute autophagy induced by EDHB. (A) HIF-1α and BNIP3 protein expression was analyzed by western blot analysis in KYSE 170 cells after treatment with 50 µg/ml EDHB for 0 h, 4 h, 12 h, 24 h and 48 h. (B) KYSE170 cells were treated with 50 µg/ml EDHB for 12 or 24 h. BNIP3, Beclin and Bcl-2 were assessed by western blotting. (C) BNIP3, LC3-I and LC3-II protein expression was analyzed by western blot after treatment with 50 µg/ml EDHB for 0 h, 4 h, 12 h and 24 h. (D) BNIP3 interacts with LC3. After treatment with EDHB for 12 and 24 h, BNIP3 and LC3 were immunoprecipitated using anti-BNIP3 or anti-LC3 antibodies. BNIP3 and LC3 were detected by western blot analysis. (E) KYSE170 cells were treated with 50 µg/ml EDHB for 4 h, 12 h and 24 h. Mitochondrial and cytosolic fractions were isolated from the treated cells and analyzed for the indicated BNIP3 and LC3 proteins by western blotting. AIF was used as a control for the mitochondrial fraction and loading. (F) Colocalization of LC3 and the mitochondrial Tom proteins was determined with immunofluorescent analysis after EDHB treatment for 24 h. Nuclei were stained with DAPI, as shown in blue. Scale bars, 10 µm;β-actin was used as a loading control.

As a prolyl-hydroxylase inhibitor, EDHB inhibits the ubiquitin-mediated degradation of HIF-1α, thus stabilizing HIF-1α protein expression. As shown in Figure 5A, HIF-1α protein significantly accumulated as the duration of EDHB treatment increased. As shown by the transcriptional expression profile, EDHB up-regulated the expression of BCL-2/EIB-19K-interacting protein 3 (BNIP3), an atypical BH3-only protein. BNIP3 is known to cause mitochondrial dysfunction and cell death. Interestingly, BNIP3 plays a central role in As_2_O_3_-induced autophagic cell death in malignant glioma cells [22]. BNIP3, Beclin and microtubule-associated protein 1 light chain 3 (LC3) are also requirement for autophagy induction and is enhanced at mitochondria [23]. These observations led us to hypothesize whether EDHB-induced autophagic cell death which is mediated by BNIP3. To determine whether the increase in BNIP3 protein is associated with autophagy, we assessed the autophagic associated protein including BNIP3, Beclin, Bcl 2 and LC3 after EDHB treatment. As shown in Figure 5B & C, EDHB induced the accumulation of BNIP3, Beclin and LC3II which facilitate the initiation of autophagy in KYSE170 cells, and reduced the expression of Bcl-2, which inhibits autophagy. To further evaluate the functional relevance of BNIP3 and LC3 on EDHB-induced autophagy, we first analyzed the protein-protein interaction between BNIP3 and LC3 using co-immunoprecipitation. Next, we assessed the expression of BNIP3 and LC3 in the cellular fraction after EDHB treatment for 4 h, 12 h and 24 h. We found that BNIP3 significantly interacted with the LC3 protein after EDHB treatment for 12 h and 24 h. Both BNIP3 and LC3 also greatly increased in the mitochondrial fraction after EDHB treatment (Figure 5D & E), indicating that the increased expression of BNIP3 and Beclin can induce autophagic cell death by localization and processing of LC3, which is incorporated in autophagic membranes upon formation. To confirm the initiation of autophagy from the mitochondria, Tom, a translocase of the outer membrane was used as a mitochondrial marker; green endogenous LC3 was partially co-localized with the red mitochondrial marker Tom20 according to immunofluorescent analysis after EDHB treatment for 12 h (Figure 5F). These observations indicated that EDHB initiates mitochondrial-derived autophagic cell death at the early stage after EDHB treatment by the regulation of BNIP3, Beclin and LC3 proteins. EDHB also affects the Bcl-2 and Beclin-1 regulatory axis that is controlled by BNIP3.

### NDRG1 was required for EDHB-induced apoptosis

**Figure 6:**
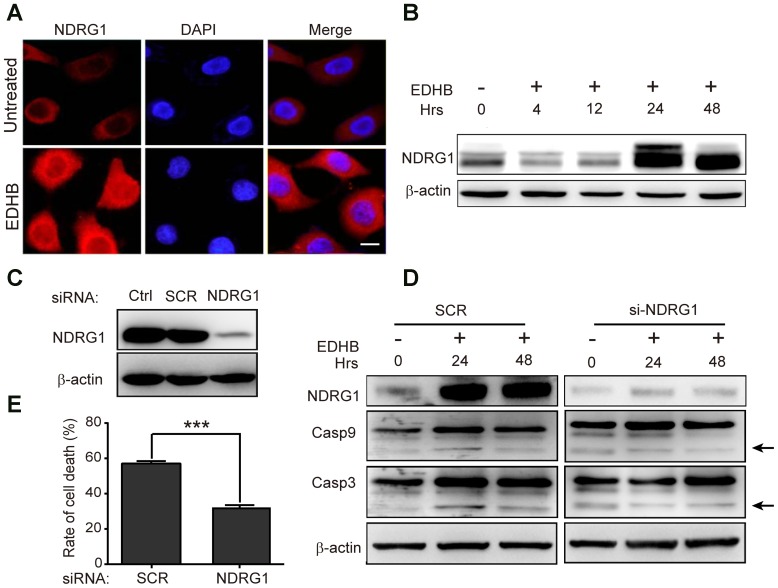
Verification of the effects of NDRG1 knock-down using western blot analysis. (A) NDRG-induced expression was determined with immunofluorescent analysis after EDHB treatment for 24 h. Nuclei were stained with DAPI, as shown in blue. (B) NDRG1 protein expression was examined by western blot analysis in KYSE 170 cells treated with 50 µg/ml EDHB for 0 h, 4 h, 12 h, 24 h and 48 h. (C) The effect of *NDRG1* gene knock-down was analyzed by western blot analysis. (D) The effect of *NDRG1* gene knock-down was analyzed by western blot analysis in KYSE 170 cells treated with 50 µg/ml EDHB for 0 h, 24 h and 48 h. Caspase-3 and caspase-9 protein expression was analyzed by western blot analysis. (E) After treatment with 50 µg/ml EDHB, the inhibition of cell proliferation following *NDRG1* gene knock-down in KYSE 170 cells was detected using the WST-8 assay. Scale bars, 10 µm; Ctrl, control; SCR, scrambled siRNA; siNDRG1, siRNA for NDRG1; Hrs, hours; ***, *P*<0.001.

As shown by the microarray analysis in Figure 6A and 6B, *NDRG1* expression was increased in response to treatment with EDHB, suggesting that NDRG1 may promote EDHB-mediated apoptosis. To confirm whether EDHB-mediated apoptosis involves NDRG1, the *NDRG1* gene was knocked down using siRNA interference technology, which was confirmed by western blot analysis (Figure 6C). We also examined whether *NDRG1* knock-down had an impact on the sensitivity of cells to EDHB. As shown in Figure 6D, *NDRG1* knock-down markedly suppressed EDHB-induced apoptosis. In addition, the activated cleaved fragments of caspase-9 and caspase-3 were significantly decreased in *NDRG1* knock-down cells after EDHB treatment for 24 h and 48 h (Figure 6E). These results indicated that EDHB increased the expression of NDRG1, subsequently inducing apoptosis; in other words, NDRG1 was required for EDHB-induced apoptosis.

## Discussion

EDHB is a natural food additive that exerts antioxidant functions and is present in many plants. Recent studies have mainly focused on the antioxidant function of EDHB and as an analog of α-ketoglutarate as well as on the role of EDHB in inhibiting collagen synthesis and in serving as a competitive inhibitor of prolyl hydroxylase activity. One study demonstrated that EDHB exerted significant protective effects and improved behavior by effectively inhibiting free radical damage and increasing the antioxidant capacity of brain tissues in a cerebral ischemic rat model [24]. In addition, another study revealed that EDHB inhibited peroxide-induced oxidative damage in astrocytes via the activation of HIF-1α protein and the promotion of downstream VEGF expression [25]. As a prolyl hydroxylase inhibitor, EDHB was also shown to inhibit collagen secretion in breast cancer cells, thus inhibiting tumor cell metastasis and reducing tumor fibrosis in a mouse breast cancer model [18].

In this study, we focused on identifying the molecular regulators involved in EDHB-induced KYSE 170 cell cytotoxicity. We found that EDHB caused concentration- and time-dependent cell death. Apoptotic cell death frequently occurs following cell cycle arrest under conditions of stress [26]. Our results revealed that EDHB significantly inhibited ESCC cell growth in a time- and dose-dependent manner through S phase cell cycle arrest, induced apoptosis and results in the loss of mitochondrial membrane permeabilization. p53 is the most extensively studied tumor suppressor governing cell cycle checkpoints and apoptosis [27]. To gain further insight into the molecular mechanisms underlying EDHB-induced cell cycle arrest and apoptosis in ESCC cells, we examined the levels of p53 and p21. We also found that caspase-9 and caspase-3 were actively cleaved along with Bax translocation and the release of cytochrome c following treatment, suggesting that EDHB induced apoptosis via the mitochondria-dependent intrinsic apoptotic pathway in ESCC cells. As a prolyl hydroxylase inhibitor, EDHB has been shown to stabilize HIF-1α protein expression by inhibiting its ubiquitin-mediated degradation [28]. HIF-1α is a key regulatory factor in hypoxic responses, and forms the HIF-1 heterodimer along with HIF-1β. Under normal physiological conditions, HIF-1α becomes ubiquitinated and degraded via a ubiquitin-dependent pathway after the amino acid residues Pro 420 and Pro 564 are hydroxylated by proline hydroxylase (PHD) [29]. The prolyl-hydroxylase inhibitor EDHB, inhibits the ubiquitin degradation pathway, protecting HIF-1α from degradation and thus stabilizing HIF-1α protein expression. In our study, EDHB did not induce alterations in HIF-1α transcript levels but significantly increased HIF-1α protein expression. The results are consistent with previous reports. Therefore, EDHB may increase HIF-1α protein expression, impacting HIF-1α-mediated downstream gene expression.

In KYSE 170 cells treated with EDHB, the expression of 43 genes increased and the expression of 9 genes decreased. An enrichment analysis revealed that these candidate genes are involved in many biological processes, including responses to organic substances, negative regulation of cellular biosynthesis processes, regulation of programmed cell death and so on. The candidate genes *NDRG1*, *CCNG2*, *VEGF*, *BNIP3*, and *AKR1C1* were therefore validated in subsequent experiments. The expression of *NDRG1* and BNIP3 was induced after EDHB treatment for 24 h. The promoter regions of *NDRG1* and *BNIP3* contain a HIF binding site that is responsible for activating *NDRG1* expression [30]. In addition, HIF-1α was shown to promote the increased expression of NDRG1 and induced U937 cell differentiation, thus terminating proliferation and causing apoptosis [31]. In another study, the small molecule L-mimosine promoted HIF-1α expression, as well as NDRG1 expression, in prostate cancer cells, thereby inhibiting prostate cancer proliferation [32]. The results of these studies indicate that hypoxia induce NDRG1 expression and promotes apoptosis. The results of the present study revealed that the treatment of KYSE 170 cells with EDHB promoted increased NDRG1 expression. *NDRG1* knock-down using RNA interference technology significantly reduced EDHB-induced apoptosis, indicating that EDHB inhibited ESCC cell proliferation and promoted apoptosis by promoting NDRG1 expression.

BNIP3 is a pro-apoptosis protein in the “BH3-only” subclass that exerts its pro-apoptotic functions by forming heterodimers with anti-apoptotic proteins through its BH3 structure. HIF-1α was shown to induce the expression of the pro-apoptotic protein BNIP3, thereby promoting apoptosis [33]. Previous studies have implicated that BNIP3 overexpression could also induce autophagic cell death by the regulation of HIF-1α. Autophagy is a normal degradative process that is stimulated in response to a variety of stress in human tumor cells after treatment with chemotherapeutic drugs [34]. Autophagy and apoptosis are often activated together in response to stress [35]. The promoter region of BNIP3 contains a HIF-1α transcription-related hypoxia-responsive functional binding element. Our results revealed that BNIP3 expression was up-regulated following EDHB treatment. Moreover, Beclin, the human Bcl-2-interacting protein, which participate in the induction of autophagy in response to a variety of stress, was regulated by BNIP3. In addition, Beclin gene transfer increases basal levels of autophagy and starvation-induced autophagy in human breast cancer cells [36], [37]. In the present study, there was considerable up-regulation of BNIP3, Beclin and LC3 and down-regulation of Bcl-2 protein expression in KYSE 170 cells after EDHB treatment, consistent with induction of autophagy. Niroop et al reported that AT101 caused cell death that was accompanied by autophagy and was mediated through the expression of the atypical BNIP3. Another study reported that up-regulation of Beclin mediated autophagic cell death in leukemia cell lines after arsenic trioxide [38], [39]. Collectively, these results suggest that up-regulation of BNIP3, Beclin and LC3 may contribute to EDHB induced autophagy and play an important role in the non-apoptotic cell death induced by EDHB. The specific mechanism by which EDHB modulates the expression of BNIP3 and Beclin remains to be investigated. However, our results corroborate previous findings and demonstrated that EDHB acts on BNIP3 and Beclin expression on intracellular trafficking of proteins, which may be the novel regulators of EDHB-induced autophagic cell death.

The expression profile microarray results presented here revealed that *AKR1C1* gene and protein expression levels were up-regulated in response to EDHB treatment. AKR1C1 is a member of the aldo-keto reductase superfamily, and this factor utilizes NADH and/or NADPH as cofactors to catalyze the conversion of aldehydes and ketones to their corresponding alcohols and also catalyzes the reduction of progesterone to 20-α-hydroxy-progesterone. *AKR1C1* is an Nrf2 target gene that plays a role in defense against internal and external oxidative and chemical stimuli. In addition, oxidative stress markers, such as 4-hydroxynonenal, act as the catalytic substrates of AKR1C1 [40]. The results of the present study showed that EDHB significantly induced AKR1C1 transcription and protein expression; however, the functional relationship between EDHB and AKR1C1 expression requires further investigation.

**Figure 7:**
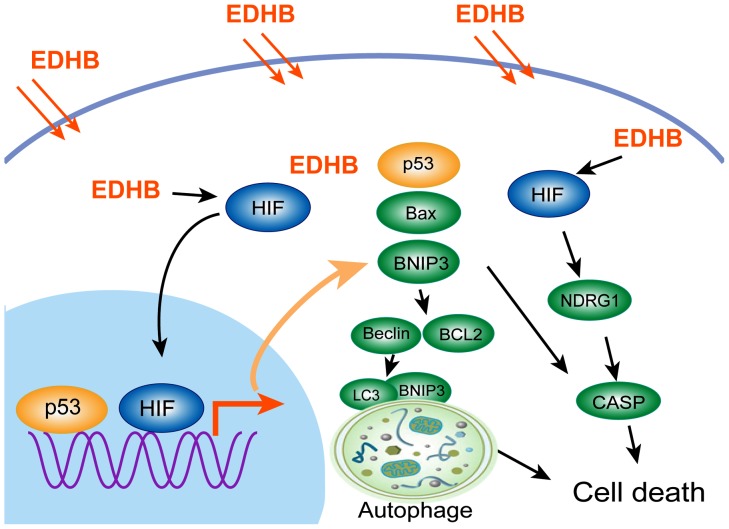
Illustration of the signaling pathway for EDHB-induced cell death. EDHB-induced apoptosis requires both BNIP3 and Beclin innate autophagy, NDRG1 associated apoptosis. The green and yellow ellipses represent up-regulated proteins, the blue ellipse represent down-regulation.

In conclusion, this study showed the effects of EDHB on cell proliferation and cell death in esophageal cancer cells. EDHB treatment led to not only apoptosis but also autophagic cell death via up-regulation of BNIP3 and Beclin and the down-regulation of BCL-2 in KYSE 170 cells. Microarray results identified a panel of differentially expressed genes, including BNIP3, Beclin and NDRG1. The increased expression of BNIP3 and Beclin initiated autophagy at an early stage, which was mediated by LC3 in KYSE 170 cells after treatment with EDHB for 12 h, ultimately resulting in the apoptosis of esophageal cancer cells. NDRG1 may be required for EDHB-induced apoptosis (Figure 7). Taken together, our results suggest that EDHB could be therapeutically beneficial for esophageal cancer.

## Supporting Information

Figure S1 **Gene expression profile analysis of EDHB-treated KYSE 170 cells.** (A) Heat map of overlapping genes. Red/green indicate an increase/decrease in gene expression relative to the universal mean for each gene. (B) GO analysis of gene expression data from KYSE 170 cells treated with 50 µg/ml EDHB. (C) Gene correlation analysis of gene expression data from KYSE 170 cells treated with 50 µg/ml EDHB.
(TIF)
