# Supplementary figures and images for: A Prolyl-Hydroxylase Inhibitor, Ethyl-3,4-Dihydroxybenzoate, Induces Cell Autophagy and Apoptosis in Esophageal Squamous Cell Carcinoma Cells via Up-Regulation of BNIP3 and N-myc Downstream-Regulated Gene-1
Source: PLoS One. 2014 Sep 18;9(9):e107204. doi: 10.1371/journal.pone.0107204 (PMC4169646; doi:10.1371/journal.pone.0107204)

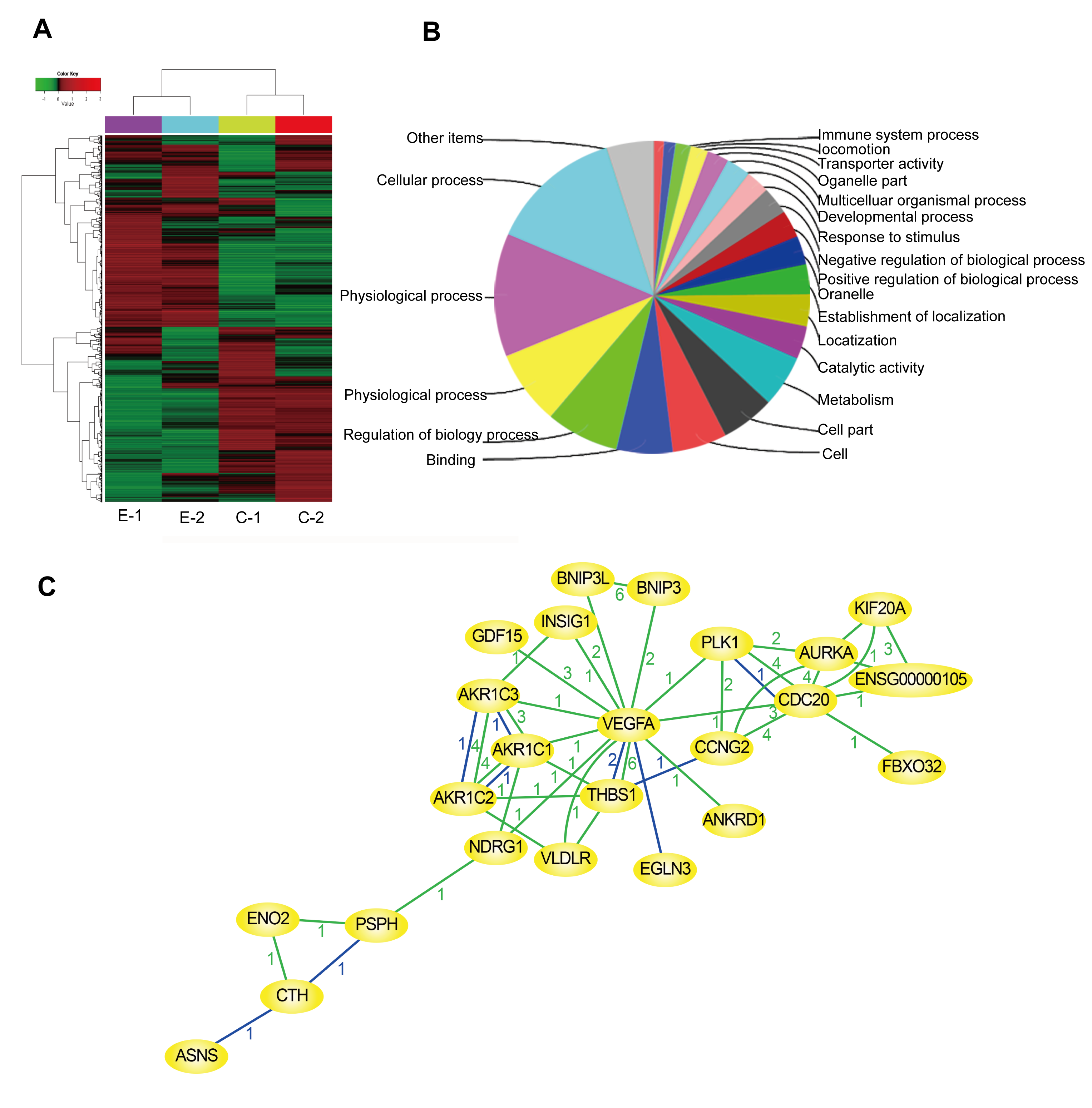

Supplement: Figure S1 — Gene expression profile analysis of EDHB-treated KYSE 170 cells. (A) Heat map of overlapping genes. Red/green indicate an increase/decrease in gene expression relative to the universal mean for each gene. (B) GO analysis of gene expression data from KYSE 170 cells treated with 50 µg/ml EDHB. (C) Gene correlation analysis of gene expression data from KYSE 170 cells treated with 50 µg/ml EDHB. (TIF) [file pone.0107204.s001.tif]
